# Afterload reduction after non-invasive vagus nerve stimulation in acute heart failure

**DOI:** 10.3389/fnhum.2023.1149449

**Published:** 2023-03-23

**Authors:** Michiaki Nagai, Keigo Dote, Masaya Kato, Shota Sasaki, Noboru Oda, Carola Y. Förster

**Affiliations:** ^1^Department of Cardiology, Hiroshima City Asa Hospital, Hiroshima, Japan; ^2^Department of Anaesthesiology, Intensive Care, Emergency and Pain Medicine, University Hospital Würzburg, Würzburg, Germany

**Keywords:** transcutaneous vagus nerve stimulation, low-level transcutaneous electrical stimulation, acute heart failure, central aortic systolic pressure, afterload

## Abstract

**Introduction:**

While central blood pressure (BP) has been recognized as a major indicator of left ventricular (LV) afterload, the reduction of central pressure decreases LV afterload and may prevent heart failure (HF) decompensation. Non-invasive transcutaneous vagus nerve stimulation (tVNS) was shown to improve cardiac function in HF patients. In this study, the relationship between active tVNS and reduction of central BP was investigated in patients with acute HF (AHF).

**Methods:**

The 22 patients hospitalized for AHF after initial stabilization (median 80 yrs, males 60%) were randomly assigned to active or sham group. For 1 h daily over 5 days, low-level transcutaneous electrical stimulation (LLTS) (20 Hz, 1 mA) was performed after attaching an ear clip to the tragus (active group) or the earlobe (sham control group). Before and after stimulation, central aortic systolic pressure (CASP), brachial systolic BP (SBP), diastolic BP (DBP) as well as heart rate (HR) were noninvasively measured.

**Results:**

No significant differences in baseline characteristics were observed between the active and sham groups. In the active group, CASP, SBP, DBP, and HR each decreased significantly after stimulation (all *p* < 0.05), whereas in the sham group, CASP, SBP, DBP, and HR each increased significantly after stimulation (all *p* < 0.05). All the changes in CASP, SBP, DBP and HR before and after stimulation were also significantly different between active and sham groups (all *p* < 0.01). There were no device-related side effects.

**Conclusion:**

In this study, the left tragus tVNS resulted in an acute afterload reduction in the elderly AHF patients. Non-invasive LLTS may be useful and safe for reducing afterload in AHF.

**Clinical trial registration:**

ClinicalTrials.gov, identifier UMIN000044121.

## Introduction

Increased loading leads to pressure- and volume-mediated remodeling of left ventricular (LV) structures (Levy et al., 1996; Kenchaiah and Pfeffer, 2004). LV hypertrophy (LVH) is an early compensatory mechanism for chronic pressure overload, which can preserve cardiac output and delay the onset of heart failure (HF). However, LV remodeling is more closely associated with decompensation and HF can occur as a result of diastolic dysfunction (Lazzeroni et al., 2016). In addition, LV remodeling is induced by activated neurohormonal pathways, including the sympathetic nervous system (SNS) and the renin-angiotensin-aldosterone system (du Cailar et al., 2000; Lopez et al., 2006). LVH has a concentric or eccentric phenotype and is associated with both HF with reduced ejection fraction (EF) (HFrEF) and HF with preserved EF (HFpEF) (Oh and Cho, 2020).

Increased wave reflex is recognized as a major hemodynamic finding of vascular aging, which is a determinant of central blood pressure (BP). Estimated central BP is shown to be superior to brachial BP in relation to target organ damage and long-term cardiovascular prognosis (Williams et al., 2006; Kario, 2010; Cheng et al., 2017), and is measured noninvasively using a variety of techniques (Cheng et al., 2017). In the PARAMETER study, angiotensin receptor-neprilysin inhibitors were superior to angiotensin II receptor blockers in reducing central systolic BP (SBP) (Williams et al., 2017). This preferential reduction of central pressure may significantly reduce LV afterload and prevent HF decompensation. Although noninvasively measured central BP represents an accurate central afterload (Chen et al., 1997; Sharman et al., 2006), to date, few studies have been conducted on changes in central BP due to therapeutic intervention in acute HF (AHF) patients.

The vagus nerve is a complex nervous system in the body, connecting vital organs such as the lungs, intestines, stomach, heart, and brain. It affects breathing, digestion, cardiac function and even mental health. Therefore, optimizing the function of the vagus nerve is thought to ameliorate target organ damage. The Xth cranial nerve has a cutaneous representation in the “Ramsay Hunt zone” located in the ear canal. *Via* Wrisberg’s intermediate nerve, cutaneous stimuli reaches the nucleus of the solitary tract (NTS) which is the main brain area of integration for vagal afferents in brainstem (Clarke et al., 1997; Ventureyra, 2000). Stimulation of “Ramsay Hunt zone” has shown beneficial effects upon seizure control (Lim, 1998; Shen, 1998). In fact, distinct vagus evoked potentials were observed after stimulation inside the tragus (Fallgatter et al., 2003). Transcutaneous vagus nerve stimulation (VNS) (tVNS) is a non-invasive, simple emergency treatment with few side effects that has spread worldwide by stimulation of the vagus nerve auricular branch of tragus (Deuchars et al., 2018). tVNS has been reported to be effective in reducing SNS activity (Clancy et al., 2014), and in Dahl salt-sensitive rats, low-level tragus stimulation (LLTS) reduced inflammatory cytokines, macrophage infiltration and myocardial fibrosis, and improved cardiac function (Elkholey et al., 2022). In addition, clinical studies have shown that LLTS significantly improved LV performance in HFpEF patients (Tran et al., 2019).

To date, no studies have examined the relationship between tVNS and afterload reduction in patients with AHF. From these perspectives, we hypothesized that active LLTS could significantly reduce central BP compared to sham stimulation in AHF.

## Materials and methods

### Study population

From June 2021 to June 2022, this study was conducted at Hiroshima City Asa Hospital (University Hospital Medical Information Network Clinical Trials Registry, UMIN000044121). Patients were considered eligible for study enrollment if they were hospitalized with AHF during this study. We defined AHF as rapid onset or worsening of symptoms and/or signs of HF. Symptoms of HF were fatigue, shortness of breath, and swollen ankles. Signs of HF were peripheral edema, elevated jugular venous pressure, and lung crackling (Mebazaa et al., 2015; Ponikowski et al., 2016). The diagnosis of AHF in each patient was based on the European Society of Cardiology 2016 guidelines for the diagnosis of HF (Ponikowski et al., 2016). For B-type natriuretic peptide used to diagnose AHF, the exclusion cut-off point is set at 100 pg/mL based on the current guidelines (Ponikowski et al., 2016). Each AHF diagnosis was made by experienced cardiologists. Consecutive eligible patients with AHF aged 40–85 years were enrolled after the study objectives were fully explained.

A sample size of 22 patients (11 in each group) would provide at least 80% power to detect this difference, at a 2-sided significance alpha level of 0.05. Inclusion criteria included (i) patients with an on-admission SBP of >100 mmHg; (ii) consenting to participate in this study. Exclusion criteria included (i) patients with multiple organ failure (possibility of unclear relationship between VNS and HF); (ii) patients in shock (possibility of excessive hypotension): (iii) patients with severe bradycardia, excluding those with a pacemaker (possibility of promoting bradycardia): (iv) patients with sepsis (possibility of excessive hypotension): (v) patients who did not consent to participate in this study.

The clinical trial protocol was approved by the Research Committee of Hiroshima City Asa Hospital (02-6-25), Hiroshima, Japan at April 6, 2023. And this study was conducted in accordance with the principles set forth in the Declaration of Helsinki. Written informed consent was obtained from all participants.

### Randomization and study procedures

#### Non-invasive vagus nerve stimulation

**FIGURE 1:**
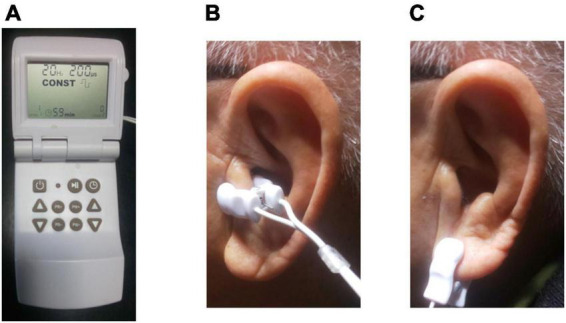
Transcutaneous electrical vagus nerve stimulation. **(A)** The device used for stimulation (Parasym, London, UK). **(B)** For active stimulation, the ear clip was attached to the left tragus, which is innervated by the auricular branch of the vagus nerve. **(C)** For sham control stimulation, the ear clip was attached to the left ear lobe, which is devoid of vagal innervation.

Patients were randomly assigned to active or sham LLTS group (1:1) after informed consent. Randomization was performed with random number table. The Parasym device (Parasym, London, UK) was used to perform stimulation (Figure 1A). In patients hospitalized for AHF after initial stabilization between 48 h and 5 days after admission, the ear clip electrode was attached to tragus in the active group (Figure 1B), while, in the sham group, the ear clip was attached to the earlobe, which is devoid of vagal innervation (Figure 1C; Lehtimaki et al., 2013; Clancy et al., 2014). A pulse width of 200 μs and a pulse frequency of 20 Hz were included in the stimulation settings (Stavrakis et al., 2015), similar to our prior report in a patient with HFpEF (Nagai et al., 2022). The stimulation amplitude was titrated to 1 mA below the discomfort threshold. For 1 h daily for 5 days, stimulation was applied by the individually trained co-medical staffs. Patients were requested to comment phenomenon related to each daily session if there were. To reduce bias, patients and investigators collecting study measurements were blinded to treatment assignments. The site of active or sham stimulation was not revealed to achieve blinding of patients.

#### Central aortic systolic pressure

Noninvasively, central aortic systolic pressure (CASP) was measured using the A-Pulse CASPro device (Health STATS, Singapore) (Ott et al., 2012). By combining calibrated radial pulse waveform data (type I calibration) and N-point moving average algorithm, the A-Pulse CASPro product was reported to share strong consistency with the invasive cardiac catheterization measurements, and its data agrees with requirement of the Association for the Advancement of Medical Instrumentation (Ott et al., 2012). We measured CASP in addition to brachial SBP, diastolic BP (DBP) as well as heart rate (HR) three times at one occasion before and after stimulation, and average values of three measurements were calculated as representative values in CASP, SBP, DBP, and HR. 5-day average values of CASP, SBP, DBP, and HR were compared before and after stimulation, and compared between two groups.

### Statistical analysis

Data are presented as median or percentages. While we used a chi-squared test for comparisons of categorical variables between the groups, Mann–Whiteney U test was performed for continuous variables at baseline. Measurements in CASP, brachial SBP, DBP and HR before and after stimulation in each group were analyzed through paired Wilcoxon signed-rank test, changes in CASP, brachial SBP, DBP and HR between the two groups were compared through Mann–Whiteney U test. A *p*-value less than 0.05 was considered statistically significant. We performed all analyses using SPSS version 11.5J statistical software (SPSS, Chicago, IL).

## Results

### Study population

**FIGURE 2:**
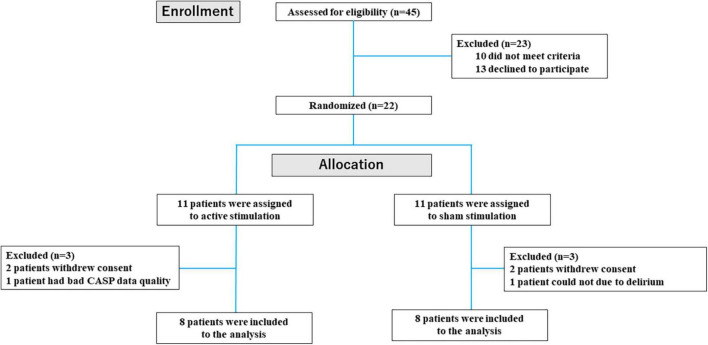
Flow diagram of participant screening and enrollment of patients.

**FIGURE 3:**
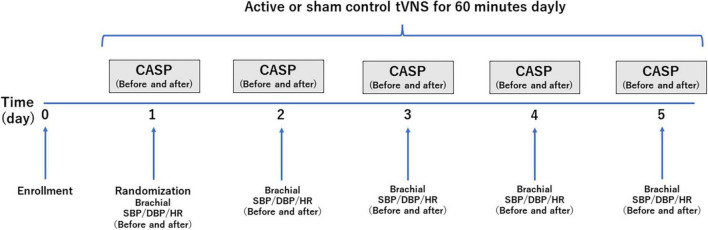
A schematic representation of the study design and timeline of hemodynamic measurements. tVNS, transcutaneous vagus nerve stimulation; CASP, central aortic systolic pressure; SBP, systolic blood pressure; DBP, diastolic blood pressure; HR, heart rate.

From June 2021 to June 2022, 45 patients were screened for eligibility, and 22 were enrolled in this study. In the 22 patients enrolled in this study (median 80 yrs, males 60%), the 11 patients (50%) were assigned to active stimulation group and the 11 patients (50%) to sham stimulation group (Figure 2). In each group, two patients withdrew from the study before stimulation. While one patient was excluded due to bad CASP data quality in active stimulation group, one patient was excluded due to delirium before stimulation procedure in sham group. Therefore, in the final analysis, a total of 16 patients with complete data were included (Figure 3). Between the 2 groups, the baseline patients’ characteristics were balanced (Table 1). Most of the patients were elderly, and HFpEF was observed in 75% of the active stimulation group and 87.5% of the sham stimulation group. Adherence to the protocol of daily stimulation was well, and all of the 16 patients were completed stimulation procedure. The stimulation amplitude has not been changed as 20 mA in both groups in each procedure over 5 days. There were no differences in BP or HR on admission between the active and sham group (134 mmHg versus 128 mmHg, respectively, *p* = 0.65 and 80 bpm versus 74b pm, respectively, *p* = 0.33). Although, in the sham group, only one person felt slight wrongness at the stimulation site, the procedure was completed without any side effects that interrupted the study.

**TABLE 1:** Baseline characteristics of the study population.

Baseline characteristics	Active (*n* = 8)	Sham (*n* = 8)	*P*-value
Age, years	79.1 (72.1; 82.1)	76.9 (70.9; 80.8)	0.13
Male, %	62.6	50.0	0.72
Body mass index, kg/m^2^	21.6 (20.3; 22.5)	22.8 (20.3; 28.0)	0.44
Ejection fraction, %	60.6 (47.2; 68.0)	63.1 (54.7; 69.8)	0.72
E/e’	23.2 (19.2; 30.1)	16.9 (13.1; 29.4)	0.44
Current smoking, %	50.0	50.0	1.0
Daily alcohol intake, %	62.5	50.0	0.72
Hypertension, %	50.0	87.5	0.23
Diabetes mellitus, %	25.0	0.0	0.44
Lipid disorder, %	25.0	12.5	0.72
Chronic atrial fibrillation, %	50.0	50.0	1.0
**Baseline laboratory values**			
Brain natriuretic peptide, pg/ml	402 (206; 555)	495 (211; 936)	0.33
Estimated glomerular filtration rate, ml/min./1.73 m^2^	35.3 (25.6; 52.8)	49.7 (36.7; 69.1)	0.13
Medication at baseline:			
ACEI use, %	0.0	12.5	0.72
ARB use, %	37.5	50.0	0.72
ARNI use, %	12.5	0.0	0.72
Beta-blocker, %	50.0	62.5	0.72
MRA use, %	25.0	12.5	0.72
SGLT2i use, %	25.0	0.0	0.44
Loop diuretics, %	62.5	87.5	0.44
Medication during admission:			
I.V. loop diuretic use, %	87.5	100	0.72
I.V. isosorbide dinitrate, %	12.5	12.5	1.0
Blood pressure and heart rate parameters:			
Systolic blood pressure on admission, mmHg	134 (120; 141)	128 (123; 137)	0.65
Diastolic blood pressure on admission, mmHg	73 (57; 84)	77 (63; 83)	0.72
Heart rate on admission, mmHg	80 (76; 96)	74 (65; 82)	0.33

Data are median and (IQR25—IQR75) or as percentages. Mann–Whiteney U test for continuous variables and the χ^2^ test for qualitative variables were used. ACEI, angiotensin-converting enzyme inhibitor; ARB, angiotensin II receptor blocker; MRA, mineral receptor antagonist; SGLT2i, sodium-glucose co-transporters 2 inhibitors; I.V., intravenous.

### Central aortic systolic pressure and other hemodynamic parameters before and after stimulation

**FIGURE 4:**
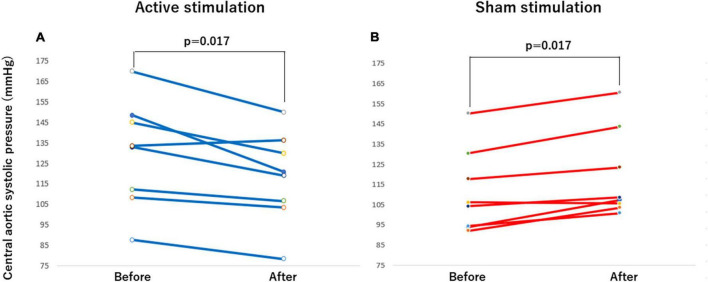
Effect of active vs sham tVNS on measures of central aortic systolic pressure. **(A)** Active stimulation. **(B)** Sham stimulation. tVNS, transcutaneous vagus nerve stimulation.

**FIGURE 5:**
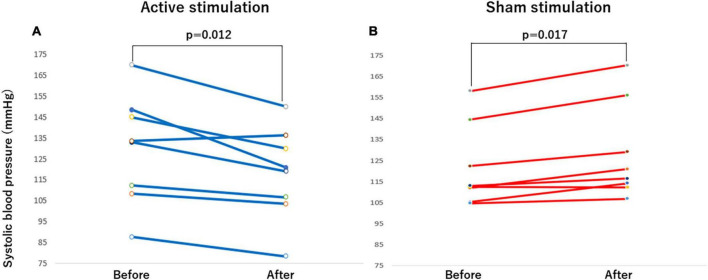
Effect of active vs sham tVNS on measures of brachial systolic blood pressure. **(A)** Active stimulation. **(B)** Sham stimulation. tVNS, transcutaneous vagus nerve stimulation.

**FIGURE 6:**
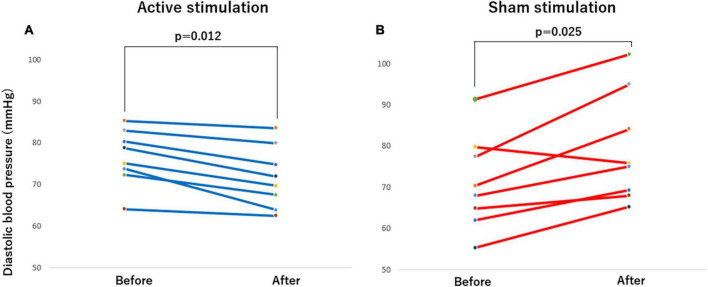
Effect of active vs sham tVNS on measures of brachial diastolic blood pressure. **(A)** Active stimulation. **(B)** Sham stimulation. tVNS, transcutaneous vagus nerve stimulation.

**FIGURE 7:**
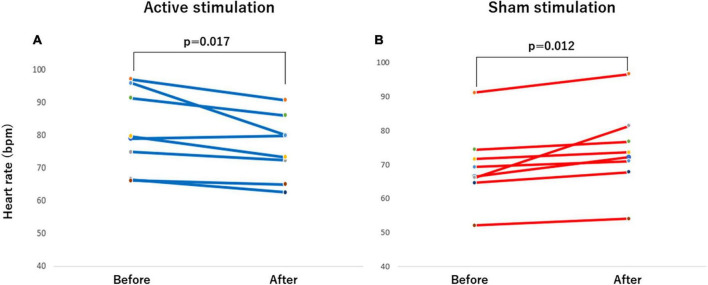
Effect of active vs sham tVNS on measures of heart rate. **(A)** Active stimulation. **(B)** Sham stimulation. tVNS, transcutaneous vagus nerve stimulation.

The outcomes of the study in the CASP and other hemodynamic parameters before and after stimulation are summarized in Table 2. Significant differences were observed in CASP, brachial SBP, DBP as well as HR before and after stimulation. In the active group, each of CASP, SBP, DBP, and HR was significantly decreased after stimulation (all *p* < 0.05) (Figures 4A, 5A, 6A, 7A), while each of CASP, SBP, DBP as well as HR was significantly increased after stimulation in the sham group (all *p* < 0.05) (Figures 4B, 5B, 6B, 7B).

**TABLE 2:** Central aortic systolic pressure (CASP) before and after stimulation.

Variables	Before stimulation	After stimulation	*P*-value
**Active stimulation (*n* = 8)**			
Central aortic systolic pressure (mmHg)	133 (109; 148)	119 (104; 135)	0.017
Brachial systolic blood pressure (mmHg)	147 (117; 160)	133 (111; 140)	0.012
Brachial diastolic blood pressure (mmHg)	77 (73; 82)	71 (65; 79)	0.012
Heart rate (beat per minute)	79 (69; 95)	77 (67; 85)	0.017
**Sham stimulation (*n* = 8)**			
Central aortic systolic pressure (mmHg)	106 (94; 127)	108 (104; 139)	0.017
Brachial systolic blood pressure (mmHg)	113 (107; 140)	119 (113; 150)	0.017
Brachial diastolic blood pressure (mmHg)	69 (63; 79)	75 (68; 92)	0.025
Heart rate (beat per minute)	68 (65; 74)	73 (69; 80)	0.012

Data are median and (IQR25—IQR75) in 5 days average values. Paired Wilcoxon signed-rank test was used.

### Changes in central aortic systolic pressure and other hemodynamic parameters between two groups

When the changes in CASP, brachial SBP, DBP, and HR before and after stimulation were compared between active and sham groups, there were significant differences in changes in CASP (*p* < 0.001), brachial SBP (*p* < 0.001), DBP (*p* < 0.01) as well as HR (*p* < 0.001) (Table 3).

**TABLE 3:** Changes in CASP between active and sham stimulation groups.

Variables	Active stimulation (*n* = 8)	Sham stimulation (*n* = 8)	*P*-value
Delta central aortic systolic pressure (mmHg)	−11.5 (−18.8; −5.03)	8.37 (4.70; 12.9)	0.0003
Delta systolic blood pressure (mmHg)	−11.5 (−14.1; −8.70)	7.87 (2.48; 11.0)	0.0002
Delta diastolic blood pressure (mmHg)	−5.10 (−6.52; −2.07)	8.60 (4.12; 13.0)	0.001
Delta heart rate (beat per minute)	−4.63 (−6.40; −1.55)	2.73 (1.95; 5.68)	0.0002

Data are median and (IQR25—IQR75) of differences of 5 days average values between before and after stimulation. Mann–Whiteney U test was used.

## Discussion

In this study, transcutaneous active stimulation of the left vagus nerve auricular branch at the tragus for 1 h was associated with an acute reduction in CASP, brachial SBP, DBP and HR. On the other hand, sham stimulation of the left earlobe resulted in an acute increase in CASP, brachial SBP, DBP and HR in patients with AHF. This first human study suggests that non-invasive LLTS is effective and safe for reducing afterload in treating AHF. This favorable effect due to tVNS on cardiac dynamics may be related to the improved sympatho-vagal balance in AHF.

### tVNS and afterload reduction

In this study, acute active tVNS resulted in a reduction in CASP in AHF patients. Increased vagus nerve activity increases muscarinic receptor activation and decreases excessive adrenergic receptor activation (Olshansky et al., 2008). tVNS has been suggested to suppress SNS induction due to inhibition of the neural activity of major ganglionated plexuses in the intrinsic cardiac autonomic nervous system (Yu et al., 2011). In post-infarction animal models, LLTS improved LV remodeling (Wang et al., 2014, 2015), cardiac autonomic remodeling (Yu et al., 2016), and reduced ventricular arrhythmia inducibility. In patients with ST-segment elevation myocardial infarction, LLTS reduced myocardial ischemia-reperfusion injury (Yu et al., 2017). On the other hand, tVNS ameliorated diastolic dysfunction in an animal model of HFpEF (Zhou et al., 2019) and acutely ameliorated LV strain in humans (Tran et al., 2019; Stavrakis et al., 2022). In a patient with HFpEF, left renal hemodynamics, right ventricular function and strain improved after 1 h of tVNS *via* the left tragus (Nagai et al., 2022).

Animal and human studies have shown that longitudinal strain was reduced by increased afterload (Donal et al., 2009; Burns et al., 2010; A’roch et al., 2012). Activation of peripheral SNS increases LV afterload *via* enhanced wave reflection (Floras, 2009; Millar et al., 2019). In this study, tVNS was associated with reduced afterload. One of the mechanisms by which tVNS reduces CASP is suggested to be suppression of SNS activity *via* tVNS that reduces the augmented wave reflection. The current findings raise the possibility that non-pharmacological interventions in tVNS that reduce sympathetic outflow improve ventriculo-aortic coupling and reduce cardiac afterload in patients with AHF.

### Active tVNS and acute hemodynamic response

In this study, brachial SBP, DBP, and HR were significantly reduced after active tVNS. In hypertensive rats, acute VNS showed beneficial effects on BP as well as HR dynamics (Plachta et al., 2014, 2016; Annoni and Tolkacheva, 2018). This acute reduction in BP was previously demonstrated when selective and non-selective VNS therapy was performed to anesthetized rats (Plachta et al., 2014, 2016). Low-intensity focused ultrasound VNS technique also reduced BP and HR in rabbits (Ji et al., 2020).

The effects of tVNS on acute hemodynamic response may be mediated by aortic depressor nerve activation, that signals the brain to enhance baroreflex and lower BP (Plachta et al., 2014). Acute noninvasive tVNS improved baroreflex sensitivity in healthy young men (Antonino et al., 2017). Anti-adrenergic, anti-inflammatory effects, activation of nitric oxide release in the heart and inhibition of the renin-angiotensin system are considered as additional mechanisms by which VNS is hypothesized to provide therapeutic effects. Suppression of central SNS outflow by activation of vagal afferent fibers leads to decreased systemic sympathetic activity, linking in decreased HR, vascular resistance, and arterial BP. In the several studies, the effect of VNS was demonstrated to decrease systemic inflammation through activation of cholinergic anti-inflammatory pathways, reducing cytokine synthesis and inflammatory responses (Tracey, 2007; Zhang et al., 2009). These mechanisms may also contribute to vascular- and cardio-protection in the long term, suggesting potential non-pharmacological HF treatments.

On the other hand, in young healthy males, a significant increase in BP and a decreasing trend in HR were observed after tVNS to the right tragus (Sinkovec et al., 2021). The evoked sympathomimetic response was indicated by a significant increase in peripheral resistance parameters and BP, and was likely a secondary baroreceptor-mediated reflex response by tVNS. The age, stimulus side and disease background differed between studies and may have been associated with contradictory findings compared to those in our study. From these perspectives, tVNS may be suitable in conditions characterized by increased SNS activity, such as AHF in our study population.

### Sham stimulation and acute hemodynamic response

Contrary to our hypothesis, in this study, sham stimulation significantly increased CASP, brachial SBP, DBP, and HR compared to before stimulation. Significant differences were observed with sham stimulation compared to active stimulation in all changes in CASP, brachial SBP, DBP and HR. An explanation for the increases in CASP, brachial SBP, DBP and HR may suggest that an increase in SNS responses to sham stimulation cannot be ruled out in AHF patients.

The earlobe is considered a reliable site for sham stimulation because of its innervation without vagus nerve fibers (Kraus et al., 2007; Frangos et al., 2015; Badran et al., 2018). Therefore, it is thought that earlobe stimulation has almost no effect derived from the parasympathetic nervous system. In fact, distinct vagus evoked potentials were seen only after stimulation inside the tragus, but not after stimulation of the earlobe (Fallgatter et al., 2003). Almost all controlled tVNS trials investigate the earlobe as a sham region, presumably the site that produces the biological activity of stimuli in terms of physiological responses. Indeed, in healthy subjects, changes in blood oxygenation level dependent (BOLD) signal evoked by earlobe transcutaneous stimulation was documented through functional magnetic resonance (fMRI) imaging studies (Butt et al., 2019). While BOLD signal activation of the NTS was not observed under the left earlobe stimulation as sham conditions (Frangos et al., 2015), left earlobe stimulation produced activation in the right insular cortex (Ic) in fMRI study.

Earlier studies supported the concept that the cardiovascular system is regulated by a central autonomic network (CAN) including anterior cingulate gyrus, amygdala and Ic (Nagai et al., 2010, 2017). Auricular tVNS activates not only the NTS but also a wide range of central nervous systems, including the CAN (Farmer et al., 2021; Hilz, 2022). Noninvasive tVNS attenuates proinflammatory cytokines and enhances antioxidant levels in brainstem and forebrain regions of Dahl salt-sensitive rats (Subramanian et al., 2020).

In patients with drug-refractory epilepsy, the right hemispheric inactivation induced an increase of high frequency of HR and BP, and the left hemispheric inactivation induced an increase low frequency of HR and BP (Hilz et al., 2001). In addition, within the Ic, a site of cardiac representation was identified (Oppenheimer et al., 1992). Tachycardia or pressor effect was observed on the stimulation of the right Ic, while bradycardia or depressor effect was significantly more common after stimulation of the left Ic (Oppenheimer et al., 1992). These results suggest that right hemisphere including the right Ic is associated with SNS activity.

In this study, sham stimulation increased both BP and HR in AHF patients, suggesting that stimulation of the left earlobe might have increased SNS activity *via* the right Ic. Further investigation is required to determine whether the hyperactivity of SNS is a physiological response due to left earlobe stimulation.

### Limitation

In this study, 6 participants dropped out of the study. It has been suggested that the presence of dropout may have influenced the study results. However, there were no significant differences in any factor when comparing the baseline characteristics of the dropout and analysis groups. Therefore, the impact of the dropout group in this study is likely to be limited. The ASCOT CAFÉ study showed that the degree of reduction in central BP by 4.3 mmHg affected prognosis in patients at high risk for cardiovascular disease (Williams et al., 2006). In the present study, active tVNS was associated with a decrease in CASP of 11.5 mmHg. Although this effect suggests a prognostic benefit, the patient demographics, baseline characteristics, study design and treatments were fundamentally different from those in the ASCOT CAFÉ study (Williams et al., 2006), limiting simple comparisons. To date, no studies have investigated the relationship between tVNS and CASP, and whether the central BP lowering effect of tVNS improves the prognosis of HF needs to be verified in future large-scale clinical trials. In this study, almost patients well responded to the initial AHF treatment, and tVNS was added to conventional treatment. Indeed, the elderly HF patients were enrolled in this study and guideline-based pharmacological treatment titrations (McDonagh et al., 2021) were not followed in the all of the patients. Therefore, tVNS may be a useful therapeutic method with few side effects even for the elderly HF patients for whom titration of guideline-recommended pharmacotherapy (McDonagh et al., 2021) is difficult and for patients in whom pharmacotherapy fails to improve the clinical outcome of AHF. At this time, these hypotheses could not be tested in this study.

## Conclusion

In this study, the left tragus tVNS resulted in an acute afterload reduction, while the left earlobe stimulation resulted in an acute afterload increase in AHF patients. Non-invasive LLTS is useful and safe for reducing afterload in AHF. Further studies will be needed to elucidate the pathophysiology underlying the relationship between tVNS and afterload reduction in relation to neurocardiology, specifically the brain-heart axis in AHF.

## Data availability statement

The original contributions presented in this study are included in the article/supplementary material, further inquiries can be directed to the corresponding author.

## Ethics statement

The studies involving human participants were reviewed and approved by the Ethics Committees of the Hiroshima City Asa Hospital (02-6-25). The patients/participants provided their written informed consent to participate in this study.

## Author contributions

MN, SS, and CF contributed to the conception of this study. MN, KD, MK, and NO participated in the patient enrollment, analysis, and interpretation of this study. MN, KD, and CF assisted and supervised the overall production of this manuscript. All authors read and approved the manuscript.
